# Identifying Marijuana Use Behaviors Among Youth Experiencing Homelessness Using a Machine Learning–Based Framework: Development and Evaluation Study

**DOI:** 10.2196/53488

**Published:** 2024-10-17

**Authors:** Tianjie Deng, Andrew Urbaczewski, Young Jin Lee, Anamika Barman-Adhikari, Rinku Dewri

**Affiliations:** 1 Department of Business Information & Analytics Daniels College of Business University of Denver Denver, CO United States; 2 Graduate School of Social Work University of Denver Denver, CO United States; 3 Department of Computer Science Ritchie School of Engineering and Computer Science University of Denver Denver, CO United States

**Keywords:** machine learning, youth experiencing homelessness, natural language processing, infodemiology, social good, digital intervention

## Abstract

**Background:**

Youth experiencing homelessness face substance use problems disproportionately compared to other youth. A study found that 69% of youth experiencing homelessness meet the criteria for dependence on at least 1 substance, compared to 1.8% for all US adolescents. In addition, they experience major structural and social inequalities, which further undermine their ability to receive the care they need.

**Objective:**

The goal of this study was to develop a machine learning–based framework that uses the social media content (posts and interactions) of youth experiencing homelessness to predict their substance use behaviors (ie, the probability of using marijuana). With this framework, social workers and care providers can identify and reach out to youth experiencing homelessness who are at a higher risk of substance use.

**Methods:**

We recruited 133 young people experiencing homelessness at a nonprofit organization located in a city in the western United States. After obtaining their consent, we collected the participants’ social media conversations for the past year before they were recruited, and we asked the participants to complete a survey on their demographic information, health conditions, sexual behaviors, and substance use behaviors. Building on the social sharing of emotions theory and social support theory, we identified important features that can potentially predict substance use. Then, we used natural language processing techniques to extract such features from social media conversations and reactions and built a series of machine learning models to predict participants’ marijuana use.

**Results:**

We evaluated our models based on their predictive performance as well as their conformity with measures of fairness. Without predictive features from survey information, which may introduce sex and racial biases, our machine learning models can reach an area under the curve of 0.72 and an accuracy of 0.81 using only social media data when predicting marijuana use. We also evaluated the false-positive rate for each sex and age segment.

**Conclusions:**

We showed that textual interactions among youth experiencing homelessness and their friends on social media can serve as a powerful resource to predict their substance use. The framework we developed allows care providers to allocate resources efficiently to youth experiencing homelessness in the greatest need while costing minimal overhead. It can be extended to analyze and predict other health-related behaviors and conditions observed in this vulnerable community.

## Introduction

The drugs [f****d] meee off for awhileee but [s**t] i gotta do what i gotta do ima addict so the [f**k] what i like to get high and yaehhhh.... facebook is lameee so you probaably wont seee me on here no more... no phoneee so ill see everyone when I see themm. i am sorry i didnt say goodbye...

Social media posting by a youth experiencing homelessness in Denver, expletives modified

### Background

Youth (persons aged between 18 and 24 years) experiencing homelessness are a vulnerable and marginalized demographic in our society. A report by the National Institute on Drug Abuse in 2019 shows that youth experiencing homelessness engage in substantially higher levels of substance use compared to housed youth [1]. Similarly, a study found that 69% of youth experiencing homelessness meet the criteria for dependence on at least 1 substance compared to 1.8% of all US adolescents [2]. The high rate of substance use among youth experiencing homelessness has a variety of proven detrimental effects, including a lower level of perceived health, depression, maladaptive coping [3], and a higher likelihood of risky sexual behaviors [4].

Despite the high rates of substance use among youth experiencing homelessness, studies have shown that they are not receiving the intervention they need [5]. Systematic inequality exists in the distribution of such opportunities for several reasons. First, intervention facilities are not equally distributed geographically [6]; there are usually fewer nonprofit facilities in economically depressed areas where youth experiencing homelessness frequently reside. Second, the breadth and depth of intervention programs are not equally distributed among substance users. Youth experiencing homelessness have limited access to care due to their lack of insurance and financial hardship [7]. Third, intervention programs, often geared toward housed adults, are not designed equally for different substance user groups [5]. Conventional intervention services may be ineffective for youth experiencing homelessness due to their transient lifestyle as well as structural and social barriers [7,8].

Social media can serve as a venue for providing efficient intervention for youth experiencing homelessness or adolescents [9]. The example social media post provided earlier demonstrates that youth experiencing homelessness may leave cues indicating their intention to initiate, quit, or relapse with substance use on their social media. The widespread and open use of social media among youth experiencing homelessness [10] provides us with the opportunity to leverage this information and identify youth experiencing homelessness at risk of substance use. Such identification is the first step for intervention programs tailored to youth experiencing homelessness, which can ameliorate the inequality in the intervention process in several ways. First, social media is not constrained by location and would allow intervention programs to improve their responsiveness to patients regardless of patients’ residence. Second, integrating a social media tool into the existing intervention efforts would be less costly to implement than traditional place-based intervention programs due to lower staffing overhead and a passive collection process, allowing for more funds to be dedicated to greater *quality care* and to servicing patients experiencing financial hardship, such as youth experiencing homelessness. Third, a social media intervention tool could provide some *flexibility* that would accommodate the transient lifestyle of youth experiencing homelessness.

### Objective

In this study, we developed a framework that can help mental health and social work professionals identify youth experiencing homelessness who may be at risk of substance use through their conversations with peers on social media. We drew from concepts in the social sharing of emotions theory [11] and social support theory [12] to develop feature sets that can be used to predict the substance use behaviors of youth experiencing homelessness. Then, we built a framework that used a variety of natural language processing (NLP) techniques to extract such features from the social media data of youth experiencing homelessness and applied multiple machine learning (ML) models to predict their substance use behaviors [13]. The goal of these models is to provide early warnings to social workers and other health professionals so that they can prioritize and provide helpful interventions to youth experiencing homelessness before their condition worsens or causes irreparable harm. Particularly, in this study, we focused on predicting marijuana use. The focus on marijuana use among youth experiencing homelessness arises from its high prevalence in this group, necessitating targeted interventions. Despite social acceptability in some areas, marijuana use among youth experiencing homelessness poses significant health and social risks. For instance, marijuana is one of the most widely used substances among youth experiencing homelessness. In a study conducted by Santa Maria et al [14], 36 (55%) of the 66 participants reported using marijuana. While decriminalization has offered some benefits, negative health outcomes have also emerged. A systematic review linked marijuana use to health issues, such as psychosis, mania, and suicide, as well as structural brain changes, impaired driving, and memory and learning impairment [15,16]. The wide prevalence of marijuana use and these negative consequences necessitate continued research to understand the specific factors influencing marijuana use among youth experiencing homelessness, allowing for the development of more effective prevention and intervention strategies.

### Mining Social Media for the General Population

Social media has become a valuable source for health informatics research [17]. The field of infodemiology [18] focuses on scanning the electronic medium (eg, the internet and social media platforms) for user-contributed health content to improve public health [19]. Particularly, a group of scholars applied advanced analytical methods to social media data to identify users’ offline health conditions and behaviors, such as depression (eg, Guntuku et al [20], Settanni and Marengo [21], Chau et al [22], Yang et al [23], and Liu [24]), suicide intention [25], poor mental health during the COVID-19 pandemic [26], and substance use [27,28].

Studies closely related to our work involve the detection of *substance use behavior* or mental risk behavior by mining social media data. Desrosiers et al [29] mined ethnic minority male adults (aged 18-25 y) SMS text messages and Facebook (Meta Platforms Inc) messages. They found that the higher negative affect in such messages was related to a higher frequency of substance use. Owen et al [30] used language models to detect depression in users of online forums. Tsugawa et al [31] used tweets to predict depression. The features used include frequencies of words, the ratio of tweet topics, the ratio of positive affect, the ratio of negative words, hourly posting frequency, tweets per day, the average number of words per tweet, overall retweet rate, overall mention rate, the ratio of tweets containing a URL, the number of users following, and the number of users followed. Hassanpour et al [27] built a deep learning model to predict drug use behavior through images and text posted on Instagram (Meta Platforms Inc). Kosinski et al [28] used the content liked by a user on Facebook to predict their substance use behavior and achieved an accuracy of 0.65. Building on this work, Ding et al [32] used the content a user likes as well as in their status updates to predict their substance use behavior. They identified topics in such content and found that certain topics are related to substance use and alcohol use. Similarly, scholars have found that keywords and topics mentioned on social media can be related to excessive drinking, both at the country level [33] and individual level [34]. For example, Marengo et al [34] mined Facebook posts and reported a positive relationship between nightlife-related and swear words and problem drinking.

While the abovementioned studies incorporated offline substance behavior, they lack 3 aspects important to our population of interest. First, they are designed for a general population or housed youth. As we discussed in the Background section, substance use among youth experiencing homelessness is much higher than that among the general population and follows a unique pattern that needs a targeted approach to understanding and interventions. This elevated substance use is symptomatic of the social and emotional challenges that these young people face. This usually means that they have different needs and priorities than other young people. These needs may be reflected in their social media interactions in terms of topics or words discussed. Second, except for a few studies (eg, the studies by Marengo et al [34] and Ding et al [32]) that examined the topics discussed in people’s social media posts, many of the studies aiming to predict substance use behavior use word embedding features that are difficult to interpret. We propose to include topic modeling in our framework so that the specific topics discussed in one’s social media posts are included as an output of our framework. Third, these studies mainly focus on one’s own narrative on social media and neglect the interactions they receive from their peers. Literature on substance use has suggested the importance of social support in reducing substance use [12]. Unique features that are afforded by social media platforms, such as comments, reactions, and other interactive features, may provide the social support needed by youth experiencing homelessness. Studies that used social media conversations to predict other mental risk behaviors also used interaction as a predictive feature [35,36]. Therefore, features captured in such interactions should be considered.

### Using Social Media to Understand the Health Behaviors of Youth Experiencing Homelessness

Similar to the general young adult population, youth experiencing homelessness use social media to stay connected with their peers and family members [37,38]. A series of studies assessing the social media use pattern and sexual health behaviors among youth experiencing homelessness helped us gain some preliminary insights into how social media is associated with sexual health behaviors among this at-risk population (eg, the studies by Young and Rice [39], Rice et al [40], and Barman-Adhikari and Rice [41]). These survey-based studies reported a relationship between the web-based social networking behavior of youth experiencing homelessness and the tendency to seek sex-related health information and engage in risky sexual behaviors, such as survival sex (exchanging sex for food, money, shelter, drugs, and other needs and wants). Rice et al [42] found that the social connections maintained on Facebook were related to the acceptability of different types of HIV prevention programs. Young and Rice [39] found that using web-based social networks for partner-seeking is associated with an increased risk of sexual behavior among youth experiencing homelessness. This trend is especially concerning for youth experiencing homelessness, as research suggests a significant portion (25%) of youth engaging in survival sex use apps to find partners [43]. The same study found that exchange sex was associated with having sexual partners who recently tested positive for HIV and an increased number of concurrent sexual partners, which has often been found to be associated with sexually transmitted infections and other risk factors [43].

Literature has shown that it is not only the structure of social network connections but also the content of such interactions (eg, email content and conversation topics) that have an impact on the health of youth experiencing homelessness [10,41,44]. Barman-Adhikari et al [10] found that youth experiencing homelessness used social media to converse about a range of topics. When they talked about topics such as drugs, drinking, or partying, they were more likely to have multiple concurrent sexual partners. Conversely, when they talked about personal goals, plans, and safe sex, they were more likely to engage in protective sexual behaviors. These findings suggest the importance of using social media as a resource for social workers to assess this hard-to-reach group and connect them with care.

The studies reviewed earlier depended on survey data, which are often flawed and difficult to collect, especially for this group. Furthermore, the social media use data obtained from the participants are indirect measures obtained solely via self-report questionnaires. Such data depend on the retrospective recall of the respondents and may not reflect the most accurate information. Nevertheless, these studies have established the connection between social media use and health-related behavior among youth experiencing homelessness. To the best of our knowledge, the only work on the drug use behavior of youth experiencing homelessness that used data collected directly from social media was conducted by Dou et al [45]. In this study, the authors combined both social media data from Facebook and survey responses from youth experiencing homelessness to predict drug use behavior [46,47]. While they used a combination of social media texts and survey data to build a model with commonly accepted levels of validity, we aimed to explore ways of predicting drug use behavior through social media data solely. We argue that this is a requirement to study this population, as it is not always feasible to obtain data via other methods, such as surveys, from youth experiencing homelessness due to their transient lifestyle. Therefore, we strove to develop a framework that can improve the performance of ML models that use only social media data. We did so by extracting more features than simply the text in the social media posts: the sentiment detected from the posts, the topics expressed in the posts, and the reactions from peers on social media.

In summary, our research objective was to develop an ML-based framework that can identify youth experiencing homelessness who may be at risk of substance use through their conversations with peers on social media. With this framework, we hope to provide early warnings to social workers and other health professionals to assist them in prioritizing and providing timely interventions to youth experiencing homelessness with the most need.

## Methods

### Ethical Considerations

Our research acknowledges the inherent privacy risks associated with social media data, which can be highly personal and sensitive [48,49]. To mitigate these concerns, we prioritized ethical data collection practices throughout the study. A cornerstone of our approach was transparency. We obtained informed consent from participants, ensuring they fully understood which data would be collected from their social media accounts and who would have access to them. The participants were provided with a total of US $50 gift cards to a food provider or a grocery store for participating in the study. This transparent approach empowers participants to make informed decisions about their data.

Following data collection, all identifiable information was meticulously removed to prevent reidentification. We used techniques such as assigning personal identification numbers to anonymize data during storage and analysis, similar to traditional research practices [50,51]. Furthermore, participants retained the right to withdraw their data at any point. We facilitated this by providing clear contact information and a web-based option for quick responses to withdrawal requests. Participants could also leverage their social media privacy settings to further restrict data access after collection.

Our research protocol strictly adhered to established ethical guidelines, including those outlined by institutional review boards for data mining on platforms such as Facebook. These guidelines emphasize minimizing risks to participants, ensuring a fair participant selection process, and prioritizing data protection throughout the research lifecycle. Our study was approved by the University of Denver Institutional Review Board (978668-1).

We also must ask ourselves how this research would be used. That is, how would individuals react if they were knowingly called out by social workers for potential substance abuse even though they did not admit to it, especially if they discovered that it was due to their social media posts? There is a probability that individuals would stop seeking services from the institutions that can help them, and they might direct other youth experiencing homelessness to not seek help from those social services. Other youth experiencing homelessness may alter their social media postings or move to “dark web” social media that are not as easily trackable. Moreover, all predictive models are subject to errors. False positives would be the bigger concern here, as youth experiencing homelessness are often ostracized for being “junkies” even if they do not abuse substances. According to Eubanks [52], predictive algorithms in social services have a history of disproportionately focusing on marginalized groups, often exacerbating the very issues they aim to solve.

The ethical implications of using social media surveillance for identifying at-risk youth are significant. As Marwick and Boyd [53] discussed, the act of surveillance can change individuals’ behavior, often leading to increased privacy measures and a decrease in trust toward institutions conducting the surveillance. Youth experiencing homelessness who are aware that their social media is being monitored might feel that their privacy is invaded, which can lead to a range of negative outcomes, including psychological distress and reluctance to engage with supportive services [54]. Furthermore, as argued by van Dijck [55], the practice of datafication, turning social behaviors into quantifiable data, can dehumanize individuals and overlook the contextual nuances of their actions, leading to misinterpretation and harm.

Given these concerns, it is crucial that any implementation of predictive models in social services includes robust ethical guidelines and continuous oversight. As discussed earlier, transparency in how data are collected, used, and shared, as well as clear communication with the affected individuals about these processes, can help mitigate some of the adverse effects. For example, youth experiencing homelessness who consented to participate should be notified of the means and frequency of their social media monitoring and the subsequent intervention and be able to choose the level and frequency of monitoring and intervention they prefer. They should also be able to terminate their participation at any time. Ensuring that social workers and other practitioners are trained in the ethical use of these tools and are sensitive to potential harm can further reduce the risk of negative outcomes.

### Recruitment and Data

We recruited youth experiencing homelessness at a nonprofit organization located in the Ballpark neighborhood of downtown Denver, a city in the western United States with a population of approximately 800,000 people in a metropolitan area of approximately 2.5 million people, between July 2017 and March 2018. Recruiters were present at the agency for >6 months, for the duration of service provision, to invite youth to participate in the study and screen them. Youth who were interested in the study were screened for eligibility and whether they owned a Facebook profile for at least a year. From youth who met eligibility criteria, we sought informed consent for participation and obtained their Facebook account information.

From these participants, we collected two types of data: (1) using our social media crawler, we collected participants’ social media conversations for the past year before they were recruited in 2017, including their Facebook posts and the comments and reactions to these posts, and (2) we asked the participants to complete a survey on their demographic information, health conditions, sexual behaviors, and substance use behaviors. Table S1 in Multimedia Appendix 1 provides survey question categories with sample questions. Specifically, participants reported whether they have used marijuana, cocaine, coke, crack, heroin, methamphetamine, or ecstasy in the last 30 days. Table S2 in Multimedia Appendix 1 provides substance use questions and answer codes. In total, we collected 135,189 Facebook conversations (including both posts and comments) from 133 participants. On Facebook, each post can also receive *reactions*, which are extensions of the Link button to allow Facebook users to share their reactions to a post. These reactions include *Like*, *Love*, *Wow*, *Haha*, *Sad*, and *Angry*. We collected such data as well.

Of the 133 participants, 3 (2.3%) had duplicated Facebook IDs and were eliminated. Table S3 in Multimedia Appendix 1 provides a summary of substance use among the remaining 130 (97.7%) participants. Out of the 130 valid participants who finished the survey, although only 13 (10%) of them indicated in their survey that they did not spend any time on a social media app on a typical day, 46 (35.4%) of them did not post anything on their Facebook timeline in the past year. This resulted in 84 (64.6%) participants with their Facebook posts and comments. We then removed the Facebook posts without any meaningful textual messages, as well as posts that had missing value in terms of the reactions they received. This resulted in 18,788 posts authored by 84 participants. In total, these posts had 19,680 comments and 80,833 reactions. Table 1 provides the summary statistics.

**Table 1:** Summary statistics.

Data sources and characteristics					Values	
**Individual-level data (cross-sectional)**						
	**From the survey (N=84 observations)**					
		Age (y), mean (SD; range)		20.58 (1.94; 18-24)		
		Male participants, n (%)		49 (58)		
		Participants attending school, n (%)		13 (16)		
		Participants currently working, n (%)		26 (31)		
		**Ethnicity, n (%)**				
			African American			18 (21)
			American Indian			2 (2)
			Asian or Pacific Islander			1 (1)
			Hispanic or Latino			12 (14)
			White			34 (41)
			>1 race or others			17 (20)
**Facebook data (time stamped)**						
	**Facebook posts (n=18,788 observations), mean (SD; range)**					
		Number of posts per person		223.7 (268.8; 1-1525)		
	**Facebook comments (n=19,680 observations), mean (SD; range)**					
		Number of comments per person		234.2 (356.1; 0-2415)		
	**Reactions (n=80,833 observations)**					
		Number of reactions per person, mean (SD; range)		962.3 (1798.9;2-12,129)		
		Like, n (%)		68,199 (84.4)		
		Love, n (%)		6975 (8.6)		
		Wow, n (%)		607 (0.8)		
		Haha, n (%)		4063 (5)		
		Sad, n (%)		961 (1.2)		
		Angry, n (%)		28 (0)		

### ML Feature Identification

To identify relevant features for building the ML framework to predict participants’ substance use behavior, we drew from literature and theories such as the social sharing of emotions theory and social support theory. We describe each feature set in the following sections.

#### Feature Set 1: Social Media Engagement

The first set of features is youth social media engagement behavior, such as the frequency of posting and the length of such posts. Studies have shown an association between social media engagement and risky behavior seeking among adolescents (see the review by Vannucci et al [56]). For example, Moreno and Whitehill [57] proposed a Facebook influence model that argued for a positive association between social media use and adolescents’ susceptibility to risky behaviors through a peer influence mechanism. The association can also be explained by the displacement hypothesis [58]; that is, social media use can replace the time spent on health-related behaviors, including in-person social interactions and physical activity. As a result, the increase in social media use among adolescents may have displaced engagement in risky behaviors, such as excessive alcohol consumption and illicit drug use [59].

#### Feature Set 2: Social Sharing of Emotions in Posts by Youth

The second set of features is the emotions or sentiments in the social media posts of youth experiencing homelessness. Social sharing of emotions refers to the verbal expression of emotions to others [11]. Social media users share their emotions to seek social support [60], enhance their emotional states [61], and regulate their emotions [62,63].

We included this feature for 2 reasons. First, the *sharing* behavior itself can be indicative of the ability to prevent or reduce unhealthy behaviors, such as substance use. Literature has shown that sharing one’s emotions allows others to provide empathy and support [11]. Such support helps people cope with stress; engage in healthy behaviors [64]; and thus reduce stress-coping responses, such as substance use [65]. Social support is also shown to be beneficial to youth experiencing homelessness; youth experiencing homelessness often seek social support from their peers, at least in the offline setting, which results in positive outcomes, such as lower rates of substance use [66].

Second, *emotions* expressed in social media content indicate substance use behavior. Research in substance use has adopted the emotion theory perspective [67] and demonstrated the causal role emotional states play in substance use behavior. For example, studies have documented a high level of emotional instability among substance abusers [68,69], as well as a connection between emotional states and substance use [70-72]. In particular, negative emotions have been observed to be associated with substance use behavior, the inability to withdraw, and the tendency to relapse. For example, studies found that negative moods are associated with a craving for alcohol [70,71]. This association may be explained by the common belief in the ability of substances to alleviate negative moods and reduce stress [73,74]. Negative emotions can also be associated with continuous use and potential relapse [70,72]. Tiffany [72] reported that negative emotional states can interfere with a conscious effort to interrupt automatic drug use behavior, therefore leading to continuous use or relapse.

#### Feature Set 3: Topics in the Posts of Youth Experiencing Homelessness

The next set of features is the topics mentioned in the social media posts of youth experiencing homelessness. There are 2 purposes for extracting topics discussed in social media posts. First, we believe that these topics are suitable features for predicting substance use behavior based on findings in related work. Psychoanalytic theory has suggested a relationship between the content of people’s conversations and people’s social behaviors [75]. Several studies have found empirical evidence of the relationship between the topics mentioned in social media content and users’ substance use behavior [32,34]. For youth experiencing homelessness in particular, Barman-Adhikari et al [10] reported an association between the topics discussed by youth experiencing homelessness on social media sites and their risky sexual behaviors. For example, talking about drugs, drinking, or partying on the web is associated with an increased likelihood of engaging in concurrent sex. This study did not examine the relationship between such topics and substance use behavior. Nevertheless, it confirmed that certain topics discussed on web-based social media can be related to offline unhealthy or risky behavior.

Second, these topics can reveal valuable information about the psychological states of the authors of the social media posts, in this case, the youth experiencing homelessness. The language one uses in both speech and writing can reveal their psychological and social states [76,77]. The users of the framework, such as social workers and researchers, can review these topics and incorporate them in follow-up interviews and surveys to better understand the situations of youth experiencing homelessness. This framework can be applied to other web-based communities to provide automatic topic extraction and summarization in social conversations.

#### Feature Sets 4 and 5: Social Sharing Interactions With Peers

Social sharing of emotions stimulates social interaction. Such social sharing interactions can strengthen social bonds and end in enhanced social integration [11]. In the case of youth experiencing homelessness, when they share their emotions and opinions on social media platforms, they also receive social interactions from their peers. For example, on Facebook, such interactions include quantitative reactions, such as *Like* and *Proud*, and qualitative comments to their posts. There is well-established literature that shows the positive impact of social support on health behaviors and health outcomes for the general population [64,78-80]. Studies that used social media conversations to predict other mental risk behaviors also used interaction as a predictive feature [35,36]. In particular, literature has suggested the importance of social support in reducing substance use [12]. Regarding youth experiencing homelessness, scholars found that they tend to seek emotional support from their peer-based networks [81]. When youth experiencing homelessness share their emotions on social media and receive support through reactions and comments*,* they may feel cared for and bonded with others, which can reduce their need for substance use. Therefore, we included both *reactions* and *comments* youth experiencing homelessness received from their peers as the last 2 feature sets in our ML framework. Particularly, for the posts of each youth experiencing homelessness, we included the number of reactions, the number of comments, and the average sentiment of all these comments. Table 2 summarizes the feature sets and the guiding theory behind including each feature set.

**Table 2:** Framework features and guiding theories.

Features		Guiding theories	Content
**Social sharing of youth experiencing homelessness**			
	Social media engagement	Facebook influence model [57] and displacement hypothesis [58] of social media	How often a youth experiencing homelessness uses the web-based platform to post content and the average length of their posts
	Social sharing of emotions	Social sharing of emotions [11] and emotion theory [67]	Each youth experiencing homelessness receives a score of overall sentiment based on all their posts
	Social sharing of topics	Psychoanalytic theory [75]	Each youth experiencing homelessness is represented by a vector indicating the proportion of each of the topics in all their posts
**Social sharing interaction with peers**			
	Peers’ reactions to the posts of youth experiencing homelessness	Social support theory [12]	Reactions such as Like, Love, and Wow to the posts of each youth experiencing homelessness, depending on the social media sites
	Peers’ comments on the posts of youth experiencing homelessness	Social support theory [12]	How actively the posts of a youth experiencing homelessness gain attention Each youth experiencing homelessness receives a score of overall sentiment based on all comments on their posts

### Feature Extraction

Among the 5 feature sets, social media engagement and reaction from peers do not need further extraction. In the following sections, we focus on how we used NLP techniques to extract sentiment and topics from the posts, as well as the sentiment from the comments.

#### Text Preprocessing

To prepare the texts in our dataset for sentiment analysis and topic analysis, we first needed to preprocess them. We first removed web links, numbers, and names because such information does not contribute to the understanding of the sentiment and topics of the texts. We did not remove punctuation because some punctuation, such as exclamation points, may carry sentiment weights. We then tokenized the texts into words and phrases and removed common stopwords, such as “and,” “the,” and “a.” The output tokens were then ready for sentiment analysis because we wanted to preserve information such as punctuation, emojis, and words in its original form. For example, “happy,” “happiest,” and “HAPPY” may have different intensities of emotion. We then performed further text preprocessing for topic modeling. First, we removed all the punctuation. Then, we converted all the texts into lowercase. After this, we performed lemmatization by reducing each word variant to its base form. Lemmatization is preferred over stemming because it could produce more readable words, as easy-to-interpret output is desirable in topic modeling. Next, we performed part-of-speech tagging for words and kept the following part-of-speech tags: nouns, verbs, adjectives, and adverbs. Finally, we believed it was important to prune candidate words to reduce noise and vocabulary size because terms with high frequency and low frequency are not very useful in topic modeling [82]. We pruned unigrams and bigrams with high and low frequencies (for example, the words that occurred in >70% or <1% of the documents) for topic modeling to reduce noise and vocabulary size. We experimented with different frequency cut-offs (between 0% and 100%, between 1% and 70%, and between 5% and 80%) in the topic modeling step and compared the results, which will be discussed later in the Topic Analysis section.

#### Sentiment Analysis

We conducted a sentiment analysis to detect the emotions and sentiments in Facebook texts [83]. We used Valence Aware Dictionary and Sentiment Reasoner (VADER), a lexicon and rule-based sentiment analysis tool, to perform sentiment analysis on our dataset of Facebook posts. To calculate the sentiment intensity expressed in each post, we first identified words in the conversation that had a sentiment orientation by using VADER’s sentiment lexicon [84]. This lexicon comprises lexical features, such as words, punctuation, phrases, and emoticons, each assigned with a valence score [85]. A valence score describes the degree of sentiment intensity, from the most negative (–1) to the most positive (+1). Then, we computed an overall sentiment score for a post by summing the valence scores of all the lexicons (including words, phrases, punctuations, and emojis) detected within the text, adjusted according to grammatical and syntactical rules, such as negation and degree intensifiers. These intensifiers are called booster words, such as “extremely” and “marginally,” which impact sentiment intensity by either increasing or decreasing the intensity. Finally, VADER normalized this score between –1 and 1. We have described this normalization process in Multimedia Appendix 2.

To evaluate the performance of VADER, we randomly selected 300 messages and manually categorized each 1 into 3 sentiment categories: positive, negative, and neutral. We then labeled the sentiment of each message predicted by VADER using the following rules: a VADER score of 0 indicated a neutral sentiment, a VADER score between 0 and 1 indicated a positive sentiment and a VADER score between –1 and 0 indicated a negative sentiment. Finally, we compared the human-annotated sentiment categories with the VADER-predicted sentiment categories and found an agreement rate of 70%. Table S4 in Multimedia Appendix 1 provides the performance of VADER classification for all messages, as well as positive, negative, and neutral messages separately.

We then ran a sentiment analysis on the 18,788 Facebook posts authored by our survey participants, as well as on the 19,680 comments made to these posts. After we calculated the sentiment scores of all Facebook posts by our participants, we aggregated such sentiment values to the individual level. Each participant is represented by a score of overall sentiment based on all their posts and a score of overall sentiment based on all the comments made to their posts.

#### Topic Analysis

To address the challenge of topic modeling short texts on social media sites, we used the author-topic model [86], which extends the latent Dirichlet allocation (LDA) method [87]. It can be viewed as aggregating messages for a user before topic modeling [88]. We did this for 2 reasons. First, LDA assumes that each document is a mixture of various topics, while a single social media post (such as a Facebook timeline update) usually only contains a single topic. Combining the posts of an author into 1 document allows the co-occurrence of multiple topics. Second, the author-topic model allows for the modeling of user interest, which suits our purpose of modeling each participant. Empirically, studies have demonstrated the superior performance of topic models learned from aggregated messages by the same user in short-text environments [89].

Because LDA does not predefine the number of topics, we needed to determine the best number of LDA topics for our dataset. We varied the number of LDA topics from 5 to 25. We used 3 commonly used criteria for selecting the optimized number of LDA-generated topics: the coherence score of topics, the rate of perplexity change (RPC), and the interpretability of topics [90,91]. First, for each number of topics, we calculated the average coherence scores of all the topics [92]. A topic is coherent if all or most of the words are related. A high average coherence score indicates better topic quality. Therefore, the number of topics corresponding to a *high* average coherence score is a good candidate for the optimized number of topics. Second, we calculated the RPC [88]. The RPC for topic number t_i_ is calculated as in the following equation:



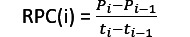

**(1)**


where P_i_ is the perplexity score [93] when the LDA model generates t_i_ topics. According to Zhao et al [90], the number of topics corresponding to the change of slope for RPC versus the number of topics is considered a good candidate for the optimized number of topics; that is, we should look for “elbows” where the RPC_i_ is smaller than RPC_i+1_. Therefore, the number of topics corresponding to a *low* RPC is a good candidate for the optimized number of topics. Third, we reviewed the top 5 representative words for each topic and interpreted them by experience [88].

Figure S1 in Multimedia Appendix 1 plots both the coherence score and perplexity change rate versus the number of topics. Figure S1 in Multimedia Appendix 1 suggests that 9 and 19 are good candidates for the best number of topics with a high coherence score and a low RPC. We reviewed the top words and interpreted them for the resulting 9 topics and 19 topics. On the basis of our review, we chose 19 topics as the best number of topics because it had a higher coherence score and gave us more interpretable representative words and underlying topics. This number was comparable to the number of topics in similar research on Facebook status updates (eg, 25 topics in the study by Wang et al [82]). We also compared the performance of different sets of word candidates, unigrams and bigrams, as inputs for topic modeling. These sets included unigram and bigrams that occurred in all documents, those that appeared in >1% but <70% of the documents, and those that appeared in >5% but <80% of the documents. The results were similar in terms of determining the optimal number of topics.

Once we determined the ideal number of topics, we represented each participant as a vector of topics, using the proportion of different topics in all their posts.

#### Text Vectorization

Finally, we developed a vector representation of each participant by vectorizing their posts. To do this, we first combined all the posts by the same participant into 1 single document. On average, each participant had 224 posts. Word embeddings or encodings are commonly used in studies that use social media data to predict health-related behaviors [94]. We used the Global Vectors for Word Representation (GloVe) embeddings, which provide pretrained word vectors [95]. These word vector representations were obtained by aggregating global word-word co-occurrence statistics that show how frequently a word appears in a context. This model is commonly used with social media texts [96] due to its pretrained nature; adaptability to domain-specific corpora; and its ability to handle sparse data, which is prevalent in the domain of social media texts. Each text was converted into a sequence of word vectors using GloVe. To handle variable length sequences, all sequences were padded to the same length, the maximum length of all sequences, by adding zeros. We combined this vector with the other 5 feature sets identified in the ML Feature Identification section.

### ML Substance Use Behavior

Using the method described in the Feature Extraction section, we represented each participant as a vector of the aforementioned features: the frequency of one’s posting, the average length of one’s posts, the average sentiment of one’s posts, the proportion of different topics in one’s posts, the average reactions one received, and the average sentiment in all the comments one received. Finally, we also included 1 more feature: the word embeddings of one’s posts. We then joined these data with participants’ survey responses so that we could use these features to predict participants’ substance use behavior.

Among the 84 youth experiencing homelessness in our final dataset, 58 (69%) were marijuana users. Table S5 in Multimedia Appendix 1 provides a summary of substance use distribution among these 84 final participants. Notably, the percentage of users for the same drug is not always consistent between the original participant pool of 130 participants and the final participant pool of 84 participants who had an active Facebook timeline in the past year. A follow-up study can investigate the discrepancy between the 2 groups of youth experiencing homelessness (those who had an active Facebook timeline in the past year vs those who did not have an active Facebook timeline in the past year) in terms of their substance use patterns. Table S6 in Multimedia Appendix 1 provides the distribution of substance use by sex group and age group.

To ensure robust performance evaluation and avoid overfitting, we used a stratified k-fold cross-validation method [97,98]. We set the number of folds to 3 due to the relatively small sample size. When splitting the data, the class distribution in the training and test sets was set to be the same as in the full dataset. The random seed for shuffling was set to a predefined value to ensure the reproducibility of our results.

We performed the ML prediction in 2 steps. Because we wanted to efficiently combine the word vector features with other numeric feature sets without losing the contextual information of the word vectors, we first used a neural network (NN) using word embeddings from the posts solely as the input to predict marijuana use. The output from this first model was then used as a prediction feature and combined with the other numeric feature sets. Subsequently, we applied all these features together to a variety of ML models. We chose this approach because it allowed us to leverage the strengths of both NNs and traditional ML models. We have described each step in detail below.

First, we used TensorFlow (Google Brain) and Keras (ONEIROS) to construct an NN model. This model first accepted pretrained GloVe embeddings of posts by a youth experiencing homelessness as the input. We used a bidirectional long short-term memory layer to capture both forward and backward sequential dependencies in the text. The long short-term memory output was passed through a dense layer with rectified linear unit activation, and the output was then flattened before making the final prediction using a sigmoid-activated dense layer.

Second, we used the output of the NN model as 1 prediction feature and combined it with the other numeric feature sets: social media engagement, social sharing of emotions, post topics, reactions, and comments. These combined features were then applied to a variety of ML models. One challenge in predicting the substance use behavior of youth experiencing homelessness is that we often have access only to very small datasets due to the transient nature of this group. On the basis of the nature of the data, we used *bagging* [99] and *ensemble learning* [100] to draw bootstrap samples from the data and perform the same estimator for each sample. The overall prediction can be obtained by simple voting. This can reduce the variance and stabilize the performance of classifiers when working with small training datasets [101]. We drew 1000 bootstrap samples from the data and performed the same estimator for each sample. The overall prediction was obtained by simple voting. We used bagging to 3 base classifiers: decision tree, logistic regression, and support vector classifier (SVC). Decision tree and SVC were suitable because they are both popular models for text classification on social media [102].

## Results

### Feature Extraction Results

In the Methods section, we discussed the NLP method of extracting sentiment and topics from the posts and comments of youth experiencing homelessness. The average sentiment of posts among the 84 participants was 0.71 (SD 0.66). Among these participants, the average sentiment of posts for marijuana users (n=58) was 0.65 (SD 0.75); while the sentiment of posts for nonmarijuana users (n=26) was 0.85 (SD 0.39). The average sentiment of comments among the 84 participants was 0.80 (SD 0.53). Among these participants, the average sentiment of comments for marijuana users (n=58) was 0.76 (SD 0.60); while the sentiment of comments for nonmarijuana users (n=26) was 0.90 (SD 0.39). These numbers reveal that nonusers have a higher proportion of posts and comments with positive sentiment scores than users.

For topic modeling, we picked 19 as the ideal number of topics based on coherence score, perplexity score, and interpretability. Table S7 in Multimedia Appendix 1 provides the top 5 topics (ranked by their frequency in the documents), their top representative words, the latent topic themes based on our interpretation, and the frequency of mention of these topics by marijuana users and nonusers. It is interesting to note that 3 (60%) of the 5 topics, namely work, swear, and female related, have been proven to be related to substance consumption among the general population [32,34].

### ML Results

As shown in Table 3, the bagged decision tree provided the most accurate prediction compared to others; therefore, it is our model choice going forward. We compared the performance of our model with a benchmark model developed by Tabar et al [103], who used survey data (such as demographic information and criminal history) to predict substance use in a similar population. In addition, we applied each of the 3 models (bagged decision tree, bagged SVC, and bagged logistic regression) using the survey data in our dataset. The features used in the survey data and the performance are reported in Table S8 in Multimedia Appendix 1. Our results showed that our feature sets outperformed the feature set of survey information when the same model was applied (with the exception of bagged SVC, when applying which the area under the curve [AUC] was 0.50 for both feature sets). Our result showed that our framework can use social media data to predict certain substance use with better performance.

**Table 3:** Performance of different machine learning models for predicting marijuana usea.

Model	AUC^b^	Accuracy	Precision	Recall	*F*_1_-score
*NN* ^c^ *and bagged decision tree* ^d^	*0.72*	*0.81*	*0.81*	*0.95*	*0.87*
NN and bagged SVC^e^	0.50	0.69	0.69	1.00	0.82
NN and bagged logistic regression	0.66	0.76	0.77	0.93	0.84
Benchmark model by Tabar et al [103]	0.72	0.69	0.73	0.79	0.76

^a^Testing size: 28.

^b^AUC: area under the curve.

^c^NN: neural network.

^d^Italicization indicates the model with the best performance in terms of AUC and accuracy.

^e^SVC: support vector classifier.

### Ensuring Fairness

Given that substance use can be disproportionally associated with certain populations, it was important to mitigate potential sex, racial, and socioeconomic biases in our framework. We followed the “fairness by design” strategy [104] to ensure fairness throughout the study. First, our team included a social work researcher, who worked to minimize the biases during data collection, model building, and result interpretation. Second, we chose to exclude the survey data from the feature selection process due to their potential to contain both explicit and implicit demographic biases. However, even with such exclusion, social media contents often contain language, topics of interest, and other contextual information that could potentially reflect a user’s demographic characteristics. These implicit cues can inadvertently introduce biases into our model. Therefore, we conducted a post hoc analysis to evaluate the model’s performance across different demographic groups, ensuring that it performs equitably. Specifically, we evaluated the false-positive rate for each sex and age segmentation. This is because we were aware of the stigma surrounding youth experiencing homelessness and drug use and wanted to identify false flags. Table 4 summarizes the evaluation results by age and sex in the test dataset, and Table S8 in Multimedia Appendix 1 summarizes the marijuana use distribution in the original dataset by age and sex. We found that the false-positive rate is slightly higher among female participants as well as participants who were aged ≥21 years. Users of this framework should deploy it cautiously and avoid overgeneralization, especially for these 2 groups.

**Table 4:** Framework performance by age and sex.

			Accuracy		Precision		Recall		False-positive rate
**Group by sex**									
	Male	0.85		0.87		0.93		0.10	
	Female	0.73		0.72		0.94		0.23	
**Group by age (y)**									
	<21	0.88		0.85		1.00		0.12	
	≥21	0.74		0.78		0.89		0.18	

## Discussion

### Principal Findings

Current studies that leverage social media data to predict users’ substance use behaviors usually neglect users’ interactions with their peers in the community, as well as the semantic meanings of their posts, such as the topics expressed. In this study, we developed a social media–based framework that applies NLP techniques and ML models to predict the substance use behavior of youth experiencing homelessness using their social media posts and interactions. We built on theories such as the social sharing of emotions and social support theory to develop an effective set of features and demonstrated the effectiveness of our framework for practical use in detecting youth experiencing homelessness at risk of using marijuana on social media platforms. Our best model reached an accuracy of 0.81 and an AUC of 0.72 when predicting marijuana use. We have observed a few notable findings.

First, we used a combination of social media posts, comments, and reactions to build a framework that can predict substance use as self-reported by the participants. Guided by theories such as the social support theory and social sharing of emotions, we developed a unique set of features from participants’ social media conversations that can be indicative of substance use behaviors.

Second, we found that the sentiment of all Facebook posts that were authored by our survey respondents was overall positive. A similar trend has been observed among housed youth [105,106]. We show that youth experiencing homelessness do not necessarily express a more negative sentiment on social media sites than their housed counterparts. Prior studies have shown that sentiment-related indicators from one’s social media texts can relate to their health characteristics, such as mental well-being, for a more general population [20,34,107-109]. Our framework provides a means to support the observation of the mental well-being of youth experiencing homelessness. It is worth noting that although we show a similar sentiment pattern between youth experiencing homelessness and other college students, it can be hard to compare the sentiment values reported by different studies due to the different sentiment analysis methods, lexicon, and scales of sentiment values used in each study. Future studies can apply sentiment analysis to Facebook conversations of both youth experiencing homelessness and their housed peers for better comparison.

Third, we also examined topics in the posts of youth experiencing homelessness extracted by our framework. The most frequent topics were related to relationships, work, swear, the female population, and lifestyle (Table S7 in Multimedia Appendix 1). We compared this list of observed topics with the list of topics reported in survey responses with an earlier study we conducted [10]. We found a few discrepancies between the 2 lists. In the survey answers, 32.6% (270/829) of participants reported talking about drugs, 26.7% (221/829) reported talking about sex, 26.2% (217/829) reported talking about school or work, 24% (199/829) reported talking about family issues, 23.9% (198/829) reported talking about being homeless, and 15.3% (127/829) reported talking about goals. While some of these topics are common, especially the topic of work, we were able to reveal a few unique topics of discussion through the use of digital trace data that would not be captured through predetermined survey questions. For example, relationship is a topic that seems to be very important for this group of young people. This likely underscores the methodological and substantive benefits of using social media data. It is interesting to note that several of these topics we found in participants’ posts, such as swear and females, are observed to be positively related to substance use among the general population [32,34]. Prior studies have shown that the topics from one’s social media texts can relate to their health characteristics, such as mental well-being. We also compared the distribution of the top 5 topics between marijuana users and nonusers in Table S7 in Multimedia Appendix 1. Overall, marijuana users tended to mention the following 4 topics more often than nonusers: relationship, work, swear, and lifestyle. Nonusers, by contrast, mentioned the female-related topic more often. While these observations highlight interesting trends, it is important to note that the differences in topic distribution may not be statistically significant. Therefore, a follow-up study with a more rigorous statistical analysis is recommended to investigate and confirm these discrepancies. Future studies can provide deeper insights into the social and psychological factors associated with marijuana use and help in understanding the underlying reasons for these differences in topic prevalence between users and nonusers.

Because our framework provides users with the capability of automatic extraction of sentiment and topics from social media conversations, one of the applications of our framework is to examine and compare the sentiment and topics expressed in social media conversations between youth experiencing homelessness and their housed peers.

Our research has several notable implications for research in mining social media for substance use behavior prediction and the practice of substance use outreach and prevention.

### Implication for Research

The major contribution of our study is the unique design of a framework that combines a series of theory-guided social media–based features that can predict the substance use behaviors of youth experiencing homelessness. The proposed feature set achieves the best AUC and accuracy compared to existing methods proposed by prior studies. These results suggest that such theory-guided features can achieve better performance over other models such as word embeddings and bag-of-words models that do not take the semantic meanings of social media conversations into account. Such results also contribute to the literature on substance use behavior. We found that the sentiment and certain topics in the posts of youth experiencing homelessness, as well as reactions and comments to these posts, can predict the marijuana use of these youth. Researchers can build on this finding to create instruments for developing a better understanding of the mental states and substance use tendencies of youth experiencing homelessness.

We also demonstrated the feasibility of mining digital trace data from social media platforms to predict the health behaviors of youth experiencing homelessness. We showed that textual interactions among youth experiencing homelessness and their friends on social media can serve as a powerful resource to predict their substance use. We proved that, without survey information, which may introduce sex and racial biases, our ML models can reach an AUC of 0.74 and an accuracy of 0.77 using only social media data.

### Implication for Practice

Substance use disorder is a significant public health concern among youth experiencing homelessness, which can potentially lead to other health-related problems (eg, risky sex behaviors, mental problems, and sexually transmitted diseases). With the increase in funding to free or subsidized treatment facilities from a local, state, or federal level [110], our work provides a foundational strategy for how these funds could be applied effectively to improve the outreach of these facilities.

After discussions with several providers in the Denver area, we believe that our work will provide an effective complementary tool for facilities’ outreach efforts. Community Alcohol Drug Rehabilitation and Education provides relapse prevention therapy services and is currently developing an outreach program. In the future, we seek to work with local providers to determine the extent to which our tool can improve their outreach efforts.

Target users of this framework include school counselors, juvenile diversion programs, shelters for people experiencing homelessness, and substance use intervention facilities. We identified 3 ways in which relevant groups, after receiving the appropriate permissions from youth experiencing homelessness, could use our framework. First, they can acquire a better understanding of the current mental state of youth experiencing homelessness by reviewing the sentiments and topics extracted from their social media pages. Second, this framework can be used as a preventive measure through the identification of the youth experiencing homelessness at risk for substance use. Third, this framework can be used for frequent monitoring and enhancing therapies designed to minimize the likelihood of relapse among youth experiencing homelessness.

Studies have shown the effectiveness of online intervention programs (eg, the study by Liang et al [111]). Our framework provides the foundation for developing web-based substance use intervention programs in social media communities. Health care agencies can work with social media companies to incorporate our framework into the platforms through which personalized interventions could be developed and distributed. Given the permissions of youth experiencing homelessness, such interventions could include appointment reminders, status monitoring services, free education, and the offering of third-party professional assistance.

### Limitations

As with all studies, this study has limitations. The first limitation of this study is the relatively small sample size compared to most social science studies. Some of this was due to almost half of the participants recruited having to be eliminated after the datasets were cleaned. While this number is considered low in laboratory research, in field research with real at-risk participants, we were happy to obtain the size that we did. While Denver is by no means a small city and unfortunately does not have a small population experiencing homelessness, it is possible that other cities with larger populations experiencing homelessness might yield larger numbers of participants. We believe that the model we created would continue to have higher levels of accuracy with more participants.

Another limitation of this study is that the data were collected between 2016 and 2017, preceding the current date. We recognize that social media platforms and communication styles can evolve rapidly [112]. However, the core issues surrounding homelessness among youth and the use of web-based platforms by youth experiencing homelessness for connection and seeking instrumental needs likely remain relevant, as evidenced by studies conducted across different time periods [37,38,113,114]. This temporal gap may affect the generalizability of our findings to the present day, but existing research suggests that the fundamental needs and behaviors of youth experiencing homelessness in web-based communication may not change as swiftly as the platforms themselves [113,114]. This consideration is critical in contextualizing our results within the evolving digital landscape.

While studying youth experiencing homelessness present difficulties due to their transient nature and distrust of outsiders [115], these data provide valuable insights. Social media analysis offers a rare window into the web-based behavior of this often-overlooked population. However, to ensure the continued relevance of our findings, further research is necessary to explore potential shifts in communication patterns among youth experiencing homelessness on social media platforms.

### Future Research

While it is hard for experts and health professionals to assess the health status of youth experiencing homelessness due to their transient lifestyle, we provide a framework that can automatically detect sentiments and opinions from social media, which can be subsequently reviewed and analyzed by experts from different research backgrounds. Future research can extend this framework to analyze and predict a variety of health-related behaviors of youth experiencing homelessness or analyze the behaviors of a more general population.

This study demonstrates the feasibility of mining digital trace data from social media platforms to predict the health behaviors of youth experiencing homelessness. Future research can look at other social media platforms, such as Instagram and TikTok (Byte Dance Ltd), and investigate other forms of digital trace data, such as images and videos.

### Conclusions

Things in my life are good and finally getting better things have been rough for me but i know i can get through the hardest of times...but i would like to thank all who have been their for me and helped me through things im coming to a new beginning and would like to still have you their with me and to renew any relations i messed up because of my drug additions i had. im now sober and well i feel great now.

Social media posting by a youth experiencing homelessness in Denver

The scourge of substance use is a major barrier to moving youth experiencing homelessness back into stable living situations. We hope that our framework will be one more tool that social service workers can use to identify those experiencing these hardships and help them receive the care they need.

## Appendix

Multimedia Appendix 1 Supplementary tables and figures.

Multimedia Appendix 2 Sentiment analysis details.

## Abbreviations

AUC: area under the curve
GloVe: Global Vectors for Word Representation
LDA: latent Dirichlet allocation
ML: machine learning
NLP: natural language processing
NN: neural network
RPC: rate of perplexity change
SVC: support vector classifier
VADER: Valence Aware Dictionary and Sentiment Reasoner
