# Supplementary material for: Identifying Marijuana Use Behaviors Among Youth Experiencing Homelessness Using a Machine Learning–Based Framework: Development and Evaluation Study
Source: JMIR AI. 2024 Oct 17;3:e53488. doi: 10.2196/53488 (PMC11528171; doi:10.2196/53488)
Supplement: Multimedia Appendix 1 [file ai_v3i1e53488_app1.docx]

# **Appendix A**

Table S1. Summary of survey question categories with sample questions

| **Categories** | **Sample Question** | **Number of Questions** |
| --- | --- | --- |
| Socio-demographic | What is your current gender identity? | 15 |
| HIV Testing and Treatment History | When was the last time you were tested for HIV/AIDS? | 25 |
| Healthcare Access and Utilization | Where do/would you prefer to receive health care services, in order of preference? | 23 |
| Homeless History | How many different times have you been without a stable place to stay? | 8 |
| Sexual-risk Behaviors | How old were you when you had sexual intercourse for the first time? | 19 |
| Recent Substance Use | During the past 30 days, on how many days did you have at least one drink of alcohol | 25 |
| Technology Access and Use | Why do you typically use social networks?  In the past three months, when you are logged on to social networks, how much time do you spend communicating with (or trying to find) prospective sexual partners? | 52 |
| Mental Health Characteristics | Over the last 2 weeks, how often have you been bothered by the following problems? (This is the 9-item questionnaire that is used to assess the level of depression) | 28 |
| Victimization Experiences | In the past 3 months, has anyone hit or attacked you without using an object or weapon? | 29 |

Table S2. Substance questions and answer codes

| **Substance** | **Survey Question** | **Answers** |
| --- | --- | --- |
| Marijuana | During the past 30 days, how many times did you use marijuana? | 1: 0 times; 2: 1 or 2 times;  3:3 to 9 times; 4: 10 to 19 times; 5: 20 to 39 times; 6: 40 or more times |
| Cocaine | During the past 30 days, how many times did you use any form of cocaine (including powder, coke, blow, or snow) but NOT crack? | Same as above |
| Crack | During the past 30 days, how many times did you use crack, including freebase or rock? | Same as above |
| Heroin | During the past 30 days, how many times have you used heroin (also called smack, junk, or China White)? | Same as above |
| Methamphetamines | During the past 30 days, how many times have you used methamphetamines (also called meth, speed, crystal, crank, or ice)? | Same as above |
| Ecstasy | During the past 30 days, how many times have you used ecstasy (also called MDMA or X)? | Save as above |

Table S3. Summary of substance use among participants

| **Drug** | **Alcohol** | **Marijuana** | **Cocaine** | **Crack** | **Heroin** | **Meth** | **Ecstasy** | **Needle injections** | **Prescription drugs** |
| --- | --- | --- | --- | --- | --- | --- | --- | --- | --- |
| **# of users** | 44 | 94 | 21 | 6 | 7 | 30 | 11 | 9 | 16 |
| **% of users** | 33.9 | 72.3 | 16.2 | 4.6 | 5.4 | 23.1 | 8.5 | 6.9 | 12.3 |

n=130, one missing value for methamphetamine

Table S4: Performance of VADER classification of 300 messages

| **Metric** | **All Messages**  (300) | **Positive Messages**  (129) | **Negative Messages**  (86) | **Neutral Messages**  (85) |
| --- | --- | --- | --- | --- |
| **Sensitivity**  **（Recall）** | 0.70 | 0.74 | 0.65 | 0.67 |
| **Precision** | 0.70 | 0.73 | 0.71 | 0.64 |
| **Specificity** | 0.85 | 0.79 | 0.89 | 0.85 |

Note: The numbers in parentheses represent the number of messages in each category. We used the micro-average approach to compute the overall recall, precision, and specificity.

Table S5. Summary of substance use among participants with active FB posts (our final data)

| **Drug** | **Alcohol** | **Marijuana** | **Cocaine** | **Crack** | **Heroin** | **Meth** | **Ecstasy** | **Needle injections** | **Prescription drugs** |
| --- | --- | --- | --- | --- | --- | --- | --- | --- | --- |
| **# of users** | 30 | 58 | 9 | 1 | 3 | 16 | 6 | 6 | 9 |
| **% of users** | 35.7 | 69.1 | 10.7 | 1.2 | 3.6 | 19.3 | 7.1 | 7.1 | 10.7 |

n=84, one missing value for methamphetamine

Table S6. Summary of marijuana use by sex and age

|  | **Marijuana User** | **Non-user** | **Total # of Observations** |
| --- | --- | --- | --- |
| **Group by Sex** | | | |
| **Male** | 0.73 | 0.27 | 49 |
| **Female** | 0.67 | 0.33 | 27 |
| **Other** | 0.5 | 0.5 | 8 |
| **Group by Age** | | | |
| **Below 21** | 0.68 | 0.32 | 47 |
| **21 and Above** | 0.70 | 0.30 | 37 |

n=84

| Table S7. Most contributing words for the top five latent topics | | | | | |
| --- | --- | --- | --- | --- | --- |
| **No.** | **Top 10 Most Contributing Words** | **Latent Topic Theme** | **Distribution within User Group** | **Distribution within Non-user Group** |  |
| #1 | really, find, tell, f**king, die, try, right, girl, happy, talk | Relationship | 38.9% | 37.5% |  |
| #2 | today, work, job, man, tomorrow, do, let, try, guy, place | Work | 22.9% | 21.5% |  |
| #3 | ass, b**h, tell, man, [N-word]s, let, even, talk, big, damn | Swear | 21.2% | 17.9% |  |
| #4 | lmaoo, baby, beautiful, sister, cute, today, morning, happy, girl, miss | Female Population | 5.1% | 6.2% |  |
| #5 | cause, real, world, keep, live, work, music, ill, soul, hard | Lifestyle | 5.4% | 4.3% |  |

Note: The topics are ordered by their occurrences in the posts. Keywords can belong to more than one topic, with different weights (probabilities). Words under the topic themes 1 and 3 are edited for the publication of this paper so as not to offend its readers.

Table S8: Accuracy of models using different features

|  | **Feature Used** | | | |
| --- | --- | --- | --- | --- |
|  | **Accuracy** | | **AUC** | |
| **Model** | **Five Feature Sets with Word Embeddings** | **Survey Information** | **Five Feature Sets with Word Embeddings** | **Survey Information** |
| NN+ Bagged Decision Tree | 0.81 | 0.56 | 0.72 | 0.44 |
| NN+ Bagged SVC | 0.69 | 0.56 | 0.50 | 0.50 |
| NN+ Bagged Logit | 0.76 | 0.69 | 0.66 | 0.52 |

Note: Because the survey information does not contain word embeddings, NN was not used to leverage word embeddings. Survey information contains the following: age, perceived health, whether the participant is working or not, whether the participant attends school or not, has the participant been in jail or not, education level, has the participant been attacked or not, race, gender, level of depression, and level of anxiety

|  |  |  |  |
| --- | --- | --- | --- |
|  | | | |
|  |  |  |  |
|  |  |  |  |
|  |  |  |  |
|  | | | |
|  |  |  |  |
|  |  |  |  |

|  |
| --- |
| **Figure S1. Coherence and Perplexity Change Rate by Number of Topics** |
