# Supplementary material for: Identifying Marijuana Use Behaviors Among Youth Experiencing Homelessness Using a Machine Learning–Based Framework: Development and Evaluation Study
Source: JMIR AI. 2024 Oct 17;3:e53488. doi: 10.2196/53488 (PMC11528171; doi:10.2196/53488)
Supplement: Multimedia Appendix 2 [file ai_v3i1e53488_app2.docx]

# **Appendix B**

We describe the process through which VADER calculates and normalizes the sentiment score of a piece of text.

VADER provides the following four scores:

1. Negative Score: This score represents the proportion of text that conveys negative sentiment. It is a measure of the amount of negative sentiment words present in the text.
2. Positive Score: This score represents the proportion of text that conveys positive sentiment. It measures the amount of positive sentiment words in the text.
3. Neutral Score: This score represents the proportion of text that is neutral, meaning it does not convey strong sentiment in either direction (positive or negative). It measures the amount of text that is neither positive nor negative.
4. In this study, we used the compound score, which is the overall sentiment of the text. The compound score is a normalized, weighted composite score that ranges from -1 (most negative) to +1 (most positive). It is calculated by summing the valence scores of each lexicon (including words, phrases, punctuations, and emojis) in the text, taking account of grammatical and syntactical rules such as negation and degree intensifiers. Then VADER uses the following formula to normalize this overall sentiment score:

$$Compound Score= \frac{sum of all valence scores}{\sqrt{{sum of valence scores}^{2}+\alpha}}$$

α is a normalization constant that ensures the scores fall within the range of -1 to 1. VADER uses a value of 15 for 𝛼, which helps scale the scores appropriately. By following these steps, VADER can provide a normalized sentiment score that ranges from -1 (most negative) to 1 (most positive). This compound score gives an overall sentiment of the text based on the individual word valences and their contextual adjustments. It is often used as a single metric to determine the general sentiment of the text.
